# Decolonising the psychology curriculum: a perspective

**DOI:** 10.3389/fpsyg.2023.1193241

**Published:** 2023-06-14

**Authors:** Peter Phiri, Sana Sajid, Gayathri Delanerolle

**Affiliations:** ^1^Research and Innovation Department, Southern Health NHS Foundation Trust, Southampton, United Kingdom; ^2^Psychology Department, Faculty of Environmental and Life Sciences, University of Southampton, Southampton, United Kingdom; ^3^Nuffield Department of Primary Care Health Science, University of Oxford, Oxford, United Kingdom

**Keywords:** decolonisation, curriculum, psychology, ethics, morality, equality, knowledge management

## Abstract

Decolonisation seeks to reverse the impact of colonisation on minoritised groups. Governments, healthcare institutions, criminal justice and education systems have procedures and protocols deep-rooted in colonisation and operate through a western lens. Decolonisation reaches beyond increasing inclusivity and aims to re-establish history through the experiences and perspective of those most affected. As with many disciplines, core theories, practices and interventions within Psychology, an ethnocentric viewpoint has been used, continuously reinstated through its curriculum. With awareness around diversification and increase in varying demands, it is important that the Psychology curriculum evolves to suit the needs of its’ users. Many recommendations for decolonising the curriculum are trivial surface changes. These involve including required bibliography from diverse minority authors within the modules syllabuses or organising a one-off lecture or workshop from a minority ethnic speaker. Some universities have also suggested that lecturers participate in self-awareness practices to ensure they understand decolonisation to appropriately address it through their teaching, whilst others have provided checklists against which they can check the inclusivity of their modules. All these alterations fail to target the root of the problem. To properly reverse the effects of colonisation within the curriculum it would be necessary to re-evaluate the Westernised history that has been retold for years and teach past events through the experiences of those who suffered. Research into how decolonisation can occur in a structured and comprehensive way is necessary to enable the redress for abolition of colonial practices on a global scale.

“When you control a man’s thinking you do not have to worry about his actions. You do not have to tell him to stand her or go yonder. He will find his ‘proper place’ and will stay in it. You do not need to send him to the back door. He will go without being told. In fact, if there is no back door, he will cut one for his special benefit. His education makes it necessary” (Woodson, 1993).

## 1. Introduction

Healthcare and academic practices and, their policies have been influenced by colonisation. It appears, colonisation and its’ related practices often prioritise the values and knowledge of western society and could be perceived as unequitable among ethnic minorities due to lived experiences with racial oppression and traumatic history (Garcia-Olp, 2018). This becomes evident through the international colonial education systems where western-focused educators, theories, textbooks, and protocols are presented as normal global phenomena (Garcia-Olp, 2018). Through such practices, native languages have been replaced with English, spiritual, native and community customs have been replaced with westernised values (Lomawaima and McCarty, 2014).

Many academic professionals distance themselves from this issue whilst others believe that simply dismissing the past will reverse the effects and impacts of colonisation (Lomawaima and McCarty, 2014). Debates surrounding inclusivity, equity, parity and diversity often ensue when decolonisation is discussed. However, this issue is not simply about making the curriculum more inclusive as this in itself does not challenge the embedded systemic racism that have been reported (Arshad, 2021; Chaussée et al., 2022). To actively target and dimmish colonisation, academics and students need to openly discuss this topic from the perspectives and experiences of all individuals whilst acknowledging the brutal history which is incorporated in many schools of thought (Arshad, 2021). This would influence societal and psychosocial dynamics to be more acknowledging of past negative experiences due to colonisation. Decolonising the curriculum is not at all about disregarding history or knowledge but relates to exploring and retelling past events through the context in which they occurred rather than from the perspective of western powers (Begum and Saini, 2019). This also demonstrates some of the biases that could go as far as impacting therapeutic benefit in some instances. Thus, acknowledging the past could improve professional trust between a patient and a mental healthcare professional.

Current literature indicates deep-rooted colonisation within academia across all levels and discussing inequalities and diversity alone does not acknowledge the deference that has existed for centuries (Gabriel and Tate, 2017; Hall and Tandon, 2017). There should be a sense of urgency to move away from trivial dialogue, deliberations, and perceptions to ensure facts are discussed in a more logical manner to reach a more transparent outcome in relation to decolonisation (Pinar, 2010; Jansen, 2018).

One of the main reasons behind the pervasiveness of colonisation is due to the perceptions and understandings of academics which can mislead the true meaning and values non-White populations practice. For example, a study exploring the perception of university lecturers towards decolonising the curriculum revealed that lecturers had very mixed and mis-informed understandings of the issue (Mashiyi et al., 2020). Some lecturers felt that there should be a shared understanding of the need to decolonise the curriculum, pondering on whether it would be a fundamental systematic change, something that would be done for research purposes, or whether decolonisation is being argued for because it is a popular topic in the media (Mashiyi et al., 2020). Some also shared the possibility of a political/racial agenda fuelling the need for decolonisation. In contrast to this, some lecturers strongly advocated for the need to involve students in extensive debates and discussions with academics to understand the issue at hand and explore practical changes which could be implemented (Mashiyi et al., 2020).

This perspective piece looks to explore the influence of colonisation on curricula, specifically in relation to the discipline of Psychology. We approach the decolonization of Psychology from a Western viewpoint, exploring current decolonization practices whilst highlighting gaps for future research.

## 2. Psychology and racism: Black psychology

Despite a growing body of evidence and knowledge base, Black psychology for example is assumed as unimportant given that the perception is psychology is a colour-blind discipline so there is no requirement to give importance to cultural paradigms (Jamison, 2021). It cannot be ignored that most psychological theories, diagnoses, and interventions were developed through an ethnocentric lens, in the West, by middle-aged White males (MacDonald, 2006). In 1851, Samuel Cartwright reported “The diseases and physical peculiarities of the negro race” (Cartwright, 1851). He claimed slaves had ‘drapetomania’, a disease which caused slaves to run away from their slave-masters whilst also attributing ‘dysaethesia aethiopica’, which affected their mind and body, causing them to have a poor work ethic (Cartwright, 1851). Cartwright stipulated that those individuals that were not slaves suffered from dysaethesia aethiopica as they “*have not got some white person to direct and take care of them*” (Cartwright, 1851, p.709). Benjamin Rush advocated for the abolition of slavery in America in the late 18th century (Rush, 1799). Through numerous pamphlets he presented the detrimental effects of slavery on mental and physical health (Rush, 1799). He also compared Black and White individuals with various illnesses to show no difference between the clinical presentations of their symptoms highlighting the lack of evidence to previous claims (Rush, 1799). Fast forward to the 20th century, we have a better understanding of the long-term impact of slavery and colonisation. In the modern day, psychologists demonstrated the presence of *post-traumatic slave syndrome* (PTSS) which is defined as a condition that occurs when multigenerational trauma is experienced by a population through slavery (Burrowes, 2019). Burrowes examined its’ effects and modern-day oppression experienced by those communities today (Burrowes, 2019). PTSS can also be explained as a consequence of persistence of racism within modern day society despite the huge political and social changes that have occurred (Burrowes, 2019). Black Psychology, a scientific sub-discipline focused on the wellbeing and experiences of individuals of African descent show further theories and subsequent evidence to report psychological experiences endeavoured specifically within this population. This field has developed various Afro-centric models of research, theories, and treatment interventions, based on individual experiences which also shows alignment to the 10 concepts of personalised medicine.

The Association of Black Psychologists (ABPsi) have worked to further the field of Black Psychology and the strategies within it as well as alter and develop several models of psychological theories and practices (Grills et al., 2018). The cofounder of ABPsi, Dr. Thomas, stated that there was a dire need for such an association to exist as he felt the American Psychological Association (APA) had not addressed the needs of the Black community and even though the Black community had been widely involved in research, resources were not shared with that community (Grills et al., 2018). Dr. Thomas also stated that the APA had failed to use its standing, resources, and capability to eradicate racism within the discipline (Grills et al., 2018). The emergence of Black Psychology forced clinicians and researchers to acknowledge that there is no universal psychological norm in terms of knowledge and practice (Grills et al., 2018).

Decolonizing the Psychology curriculum from this perspective is vital to incorporate the contribution of various Black psychology scholars and academics. By recognizing the history and perspectives of Black psychology, a more inclusive environment can be developed for African students.

## 3. Racism within the psychology curriculum

The Chief Executive of the British Psychological Society (BPS), Sarb Bajwa, wrote that the BPS is *‘institutionally racist’* further adding to the current debate in relation to making a permanent change to decolonise the psychology curriculum (Bajwa, 2020). Students and staff across the globe have protested calling for urgent action to combat decolonisation within academia in particular to actively promote anti-racism policies and practices (de Oliveira Andreotti et al., 2015; Bhatia, 2017; Gillborn et al., 2021). However, it is continually dismissed that the academic curricula, especially within Psychology, represent a Western-centric worldview (de Oliveira Andreotti et al., 2015; Bhatia, 2017; Gillborn et al., 2021). Decolonising the psychology curriculum is an important step in overcoming obstacles to global psychological theory, research, and practice.

Research indicates minority ethnic applicants applying for psychology doctorate programmes are less likely to meet the selection criteria compared to their White counterparts (Scior et al., 2007). A qualitative study found that all their participants identified and recognised the *whiteness* of their curriculum, which discussed those experiences attributed mostly to the White population and a lack of palpable examples showing the experiences from those from ethnic minorities. Through decolonisation, there could be a significant decrease of attainment gap between White and minority ethnic groups, whilst student retention and engagement rates increase (Chaussée et al., 2022).

Ethnic minority groups have differing cultures and traditions in comparison to White populations, thereby the requirement to understand, evaluate and address the psychological needs would be different (Gillborn et al., 2021). For example, a White person is more likely to report psychological distress or seek for mental health support than someone from an ethnic minority group. Similarly, an individual from an ethnic minority group is more likely to express their views on racism than their White counterpart. Gillborn and colleagues reported, their study participants found Western society was positioned as the ideal norm within psychology, with the majority of research and theories being based on White -population-based experiences (Gillborn et al., 2021). This furthers the importance and need for decolonising the psychology curriculum due to the damaging ideologies and single-sided experiences taught within (Bhatia, 2017; Gillborn et al., 2021). Mental health attributes, thoughts and prognoses varies individually and across ethnicities (Bhatia, 2017; Gillborn et al., 2021).

## 4. Racism within medicine and psychiatry

Bracken and colleagues noted that it is becoming more apparent that racism and colonialism is engrained within the Medicine, and more specifically Psychiatry, curricula and practices (Bracken et al., 2021). Evidence-based medicine is a key composite to promote the use of high-quality scientific evidence to develop optimal clinical practices. Despite this, it is not always used by institutions, as indicated by Wong and colleagues (Wong et al., 2021). These disciplines are immensely entangled with organisational, political, and commercial interests and often, interpretation is not quantitatively led (Goldacre, 2014; Wong et al., 2021). It has been suggested that the first step towards decolonising the medical and psychiatric curriculum is to recognise any biases and critically evaluate practices within these disciplines (Goldacre, 2014; Wong et al., 2021).

Positive and meaningful change can incur when there is an in depth understanding of colonialism and its impact on the modern-day populations (de Oliveira Andreotti et al., 2015; Bhatia, 2017; Gillborn et al., 2021). This is a significant component to improve population sciences and epidemiological approaches used to combat communicable and non-communicable diseases. Various Black scholars, including anthropologists, psychiatrists, and of course psychologists, have explored and introduced culturally informed psychological theory (Tyler et al., 2022). Research in anthropology has shown that *whiteness* directly reflects into racism, facilitating and fuelling Black trauma (Drake, 1980; Tyler et al., 2022). Black Psychology has enabled the advancement of these theories, understanding the experiences and exposures of individuals of Black heritage (Drake, 1980; Tyler et al., 2022).

Wong et al. (2021) propose that through the decolonisation of medicine, the discipline itself would become more humanised than medicalised, with a broader and responsive understanding of health, illness, and treatment (Wong et al., 2021). Whilst training clinicians and practitioners to be culturally responsive is important, developing their understanding in how structures and institutions disadvantage communities is vital as well (Shtasel et al., 2019). The history of institutional racism and White privileges as well as the need for cultural competency should be taught to all trainees so they understand the factors which developed their disciplines across time (Shtasel et al., 2019).

## 5. Previous work: frameworks

**Figure 1:**
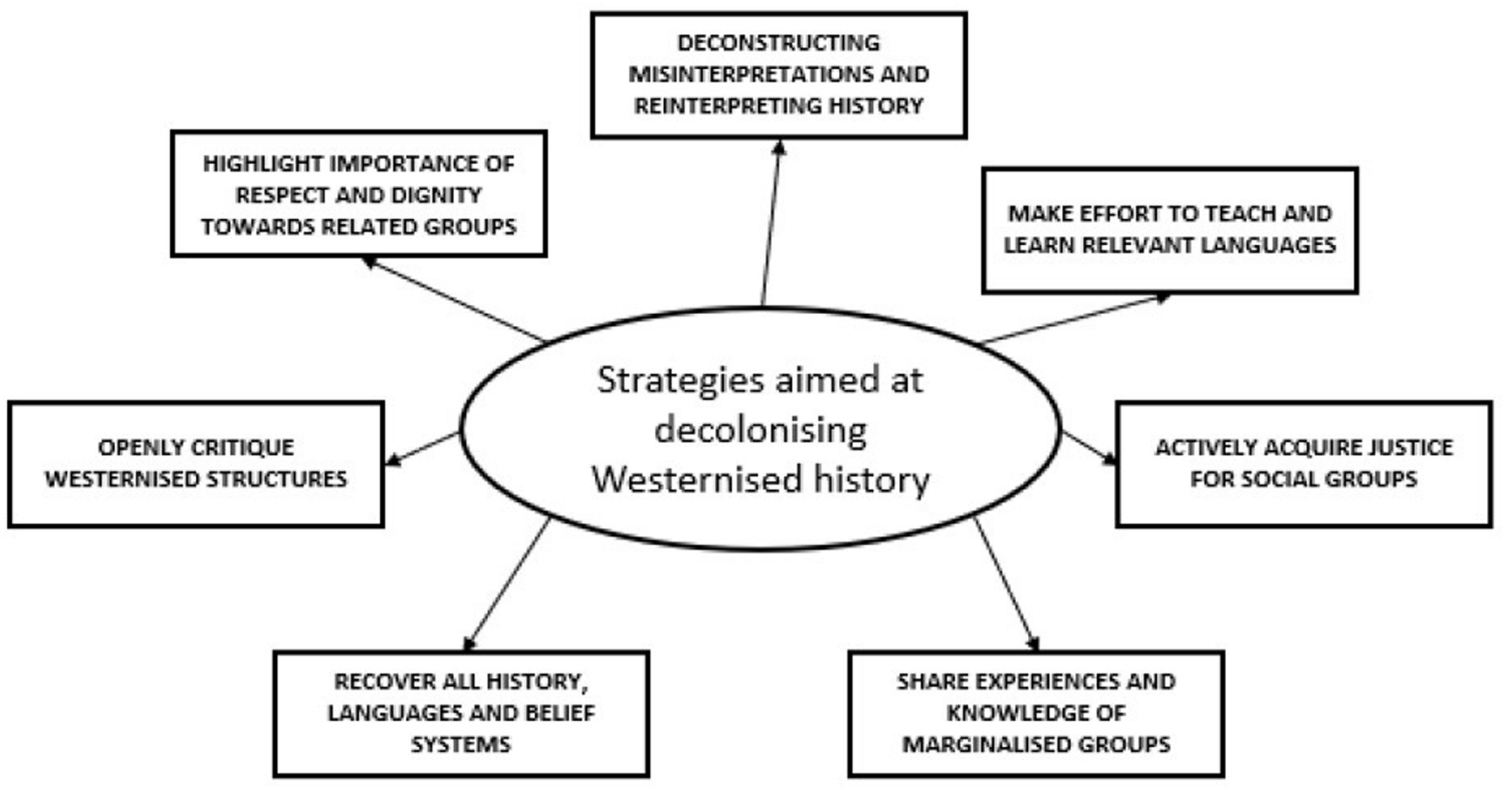
This figure outlines the seven strategies aiming to decolonise the Westernised narration of history.

Smith stated that the histories and experiences of ‘indigenous people’, referring to those belonging to minority ethnic groups, have been represented through the lens of a colonised world (Smith, 1990). Seven strategies were proposed by Smith which aimed to decolonise the Western narration of history (Figure 1) (Kottmann et al., 2019). It is important to note that this was not aimed at academia but rather colonisation in general.

Research by Abu Moghli and Kadiwal (2021) found that universities tend to engage in ‘soft reforms and surface changes to decolonise the curriculum whilst it seemed that students pushed for more radical, deep-rooted alterations. Two key strategies used by universities were identified. The first was the diversification of the curriculum, which meant to include accounts of minority ethnic work within taught curricula (Abu Moghli and Kadiwal, 2021). This was most implemented by assigning book chapters or journal articles by ethnic minority authors. There was also the off chance that a guest lecturer from an ethnic minority background would be invited or students would be obliged to take a ‘non-British module’ to make the course appear more diverse with the supposed aim of decolonisation (Bhambra et al., 2018; Abu Moghli and Kadiwal, 2021). It is important for the curriculum to reflect the cultural context under which history occurred but also incorporate indigenous knowledge and values relevant to that society (Chakawa and Maree, 2021). The second strategy was to alter staff structures in order for there to be more of an ethnic mix within the universities. Most universities target the issue of decolonisation by merely increasing access to resources for minority ethnic groups to equip them with extra skills and knowledge; these implementations do not combat the deep structural components which fuel colonisation leading to a continuation of this issue (Bhambra et al., 2018; Shepherd, 2020).

The Rhodes Must Fall Movement originated from the University of Cape Town for the removal of a statue commemorating Cecil John Rhodes, the British imperialist which sparked protests within other educational institutions across South Africa and internationally (Kgari-Masondo, 2020). The reason for such an objection was due to the values and ideologies held by Cecil Rhodes. He believed that the British rule should continue across South Africa, and he considered the South African population to be inferior to the British (Kgari-Masondo, 2020). Whilst this movement initially focused on removing the statue of one oppressive individual, it quickly spread to encompass the wider issues of institutional racism and colonised curriculums in educational institutions (Kamanzi, 2015). Following this event, students at the University of Oxford shared their frustration at the institution’s resistance to remove their own statue of Rhodes and have now begun their own movement: ‘Rhodes Must Fall in Oxford (RMFO)’ (Chaudhuri, 2016). However, Oriel College, Oxford backtracked their decision to remove the statue of Rhodes citing legal, regulatory, and financial challenges despite independent commission and campaigners’ recommendations for its removal (Mohdin, 2021). Oxford students have created blogs, webpages, and podcasts with the aim to decolonise the university by tackling colonial iconography, rebuilding the colonised curriculum and address the unequal treatment and support for minorly ethnic staff and students (Rhodes Must Fall in Oxford, 2016). In the summer of 2020 during the pandemic and Black Lives Matter protests that swept the globe following the brutal murder of George Floyd, in the USA, protestors in Bristol, UK tore down a statue of Edward Colston, a slave trader and rolled it into the nearby harbour. This action divided public opinion with some believing this action was censoring history and the then home Secretary Priti Patel stating that it was ‘utterly disgraceful and undermined anti-racism protests’ (Sky News, 2020).

### 5.1. Current practices of decolonisation within psychology

**Figure 2:**
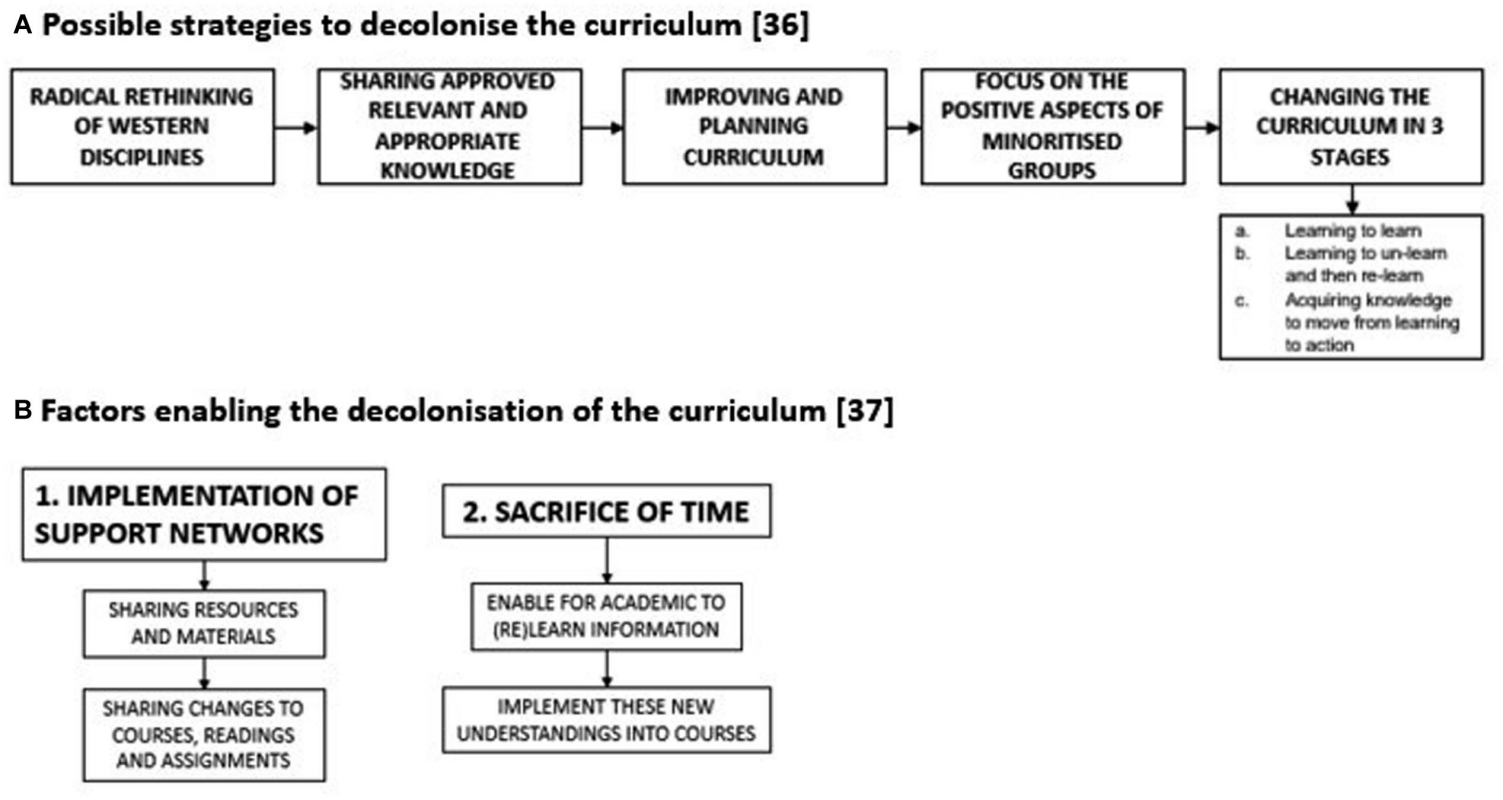
**(A)** Possible strategies to decolonise the curriculum (Le Grange, 2018) and **(B)** Factors enabling the decolonisation of the curriculum (Moncrieffe et al., 2019).

Le Grange looked at decolonising the curriculum within South Africa and suggested five implementations which could enable this illustrated in Figure 2A (Le Grange, 2018). Moncrieffe et al. proposed two factors which will enable decolonisation the curriculum (Figure 2B) (Moncrieffe et al., 2019).

## 6. APA

To address issues of the colonial legacy within psychology, the American Psychological Association (APA) Office of International Affairs organised a conference with U.S. and South African partners (Adams, 2019). The ‘Toward a Decolonial Psychology: Theories from the Global South’ conference, highlighted the need to confront such colonialism within mainstream knowledge and education (Adams, 2019). In addition to this, APA also hosted a webinar again focusing on colonial psychology (Hulme, 2018).

## 7. BPS

The British Psychological Society (BPS) produced a book titled ‘Culture Across the Curriculum: A Psychology Teacher’s Handbook’ designed to address the challenges of colonialism within Psychology (The BPS, 2020). Its aim is to provide advice and resources for staff who wish to highlight colonialism and effectively integrate the role of culture within their lessons (The BPS, 2020). The BPS also organised a webinar which discussed how decolonisation can redevelop cultural understandings of those individuals who were undermined by history (The BPS, 2020).

## 8. Other resources

BMEPsychology is a website aimed at promoting awareness about colonised curriculums whilst actively providing resources academics can use to decolonise their teaching (Jankowski, 2016). There is a huge pool of research, resources, webinars, and other activities, all focused at decolonising the psychological curriculum which are free to access and use. The University of London’s Decolonising SOAS Working Groups have developed a ‘Learning and Teaching Toolkit for Programme and Module Convenors’ (University of London, 2018). It explains what racism and decolonisation is and how these have implications within higher education settings (University of London, 2018). The toolkit goes on to explain how academic staff can decolonise their curricula and pedagogies, through examples and case studies (University of London, 2018). Advance HE have developed a ‘self-evaluation framework’ which academics can use to identify their own stance in terms of diversity and equality within the curriculum (Advance HE, 2010). University College London (UCL) have also created an ‘Inclusive Curriculum Health Check’ which can be used to assess the programme content to ensure it is accommodating for all students, including their experiences and backgrounds (University College London, 2018).

## 9. Discussion

Decolonising the Psychology curriculum would be a complex and long-term task involving re-evaluation of a number of other levels of education that may need to include primary and secondary schools. Plausible affiliations to decolonising a curriculum starting from the early education stages would further aid the Psychology domain. The global understanding of mental health, its’ causal-effects and treatments are closely linked to ethnocentric values, cultural norms, and personal belief systems. It is therefore challenging to use a theoretical basis. The recommendations discussed above mainly focus on small changes towards inclusivity within the psychology curriculum, with very minimal discussion of grass-root issues associated with colonialism as a discipline in its own right. A full-scale re-evaluation of the Psychology domain, including its theories, frameworks, treatments, and training using a more eastern and western cultural approach. Organisations such as APA, BPS and the British Association for Behavioural and Cognitive Psychotherapies (BABCP) should be leading advocacy for these changes. Likewise, educational institutions rather than ‘whitewashing’ contributions from diverse contributors across all disciplines should strive to ensure revised curriculum that acknowledges both scientific and historic contributions of the significant other. Research on the impact and effectiveness of decolonisation frameworks and approaches is warranted. To overcome such obstacles, increased collaboration between researchers and scholars across the globe is needed which would involve sharing knowledge, ideas, and perspectives to promote cultural sensitivity and understandings in psychological research and practice.

## Data availability statement

The original contributions presented in the study are included in the article/supplementary material, further inquiries can be directed to the corresponding author.

## Author contributions

PP and GD conceptualised the idea. PP and SS wrote the first draft. All authors contributed to the article and approved the submitted version.

## Conflict of interest

The authors declare that the research was conducted in the absence of any commercial or financial relationships that could be construed as a potential conflict of interest.

## Publisher’s note

All claims expressed in this article are solely those of the authors and do not necessarily represent those of their affiliated organizations, or those of the publisher, the editors and the reviewers. Any product that may be evaluated in this article, or claim that may be made by its manufacturer, is not guaranteed or endorsed by the publisher.
